# Prescription of cannabinoids in psychiatry: (how) do we cross that door?

**DOI:** 10.1192/j.eurpsy.2023.107

**Published:** 2023-07-19

**Authors:** A. Batalla

**Affiliations:** Psychiatry, UMC Utrecht, Utrecht, Netherlands

## Abstract

**Abstract:**

Cannabis policy liberalization has increased the availability of cannabis products for medical and recreational purposes, following a growing public demand. The large number of cannabinoid users reporting self-medication for several mental health-related problems and the limited medical indications for cannabis prescription have led to prescription dilemmas and confronted views between patients and clinicians. This discrepancy in perspectives grows together with a huge terminological confusion regarding medical cannabis, as some subjects use recreational products for medical purposes. In this session we will outline the current controversies of cannabis prescription in psychiatry and discuss how to deal with (il)legitimate patient needs and prescription barriers. Finally, we will discuss research approaches that could shed light on this controversial topic.

**Disclosure of Interest:**

None Declared

